# Effects of Manganese Exposure on Olfactory Functions in Teenagers: A Pilot Study

**DOI:** 10.1371/journal.pone.0144783

**Published:** 2016-01-14

**Authors:** Emilia Iannilli, Roberto Gasparotti, Thomas Hummel, Silvia Zoni, Chiara Benedetti, Chiara Fedrighi, Cheuk Ying Tang, Christoph Van Thriel, Roberto G. Lucchini

**Affiliations:** 1 Interdisciplinary Center "Smell & Taste", Department of Otorhinolaryngology, TU Dresden, Dresden, Germany; 2 Neuroradiology Unit, University of Brescia, Brescia, Italy; 3 Occupational Medicine, University of Brescia, Brescia, Italy; 4 Radiology & Psychiatry, Icahn School of Medicine at Mount Sinai, New York, NY, United States of America; 5 Neurotoxicology and Chemosensation, Leibniz Research Centre for Working Environment and Human Factors at the TU Dortmund (*IfADo*), Dortmund, Germany; 6 Preventive Medicine, Icahn School of Medicine at Mount Sinai, New York, NY, United States of America; Northeastern University, UNITED STATES

## Abstract

Long-term exposure to environmental manganese (Mn) affects not only attention and neuromotor functions but also olfactory functions of a pre-adolescent local population who have spent their whole life span in contaminated areas. In order to investigate the effect of such exposure at the level of the central nervous system we set up a pilot fMRI experiment pointing at differences of brain activities between a non-exposed population (nine subjects) and an exposed one (three subjects). We also measured the volume of the olfactory bulb as well as the identification of standard olfactory stimuli. Our results suggest that young subjects exposed to Mn exhibit a reduction of BOLD signal, subjective odor sensitivity and olfactory bulb volume. Moreover a region of interest SPM analysis showed a specifically reduced response of the limbic system in relation to Mn exposure, suggesting an alteration of the brain network dealing with emotional responses.

## Introduction

Elevated manganese (Mn) exposure during pre/early adolescence is associated with deficits in attention, neuromotor, and olfactory function in children and elderly [1]. This occurs essentially in areas with industrial contamination [2]. The route of intoxications can be hand-to-mouth [3], via inhalation/ingestion of soil particulates [4] but also trough consumption of contaminated locally grown foods [5] and drinking water [6, 7].

Upon these findings several crucial aspects still need to be addressed like the role of Mn exposure timing on adolescent executive behavior in order to define critical developmental windows of life spam of susceptibility. In environmental health, it is widely believed that fetal life, infancy and pre-adolescence are critical exposure windows for chemical toxicants, due to processes involved in the growth and differentiation of the central nervous system that accelerate during these life stages [8]. Of specific interest are the lesion patterns produced by Mn exposure in brain areas associated with executive function, behavior, and self-regulation in adolescence.

In Italy some areas in the province of Brescia have a long history of ferroalloy plant activity. In Valcamonica, a pre-alpine valley, 3 ferroalloy plants have been active for about a century until 2001 while in Bagnolo Mella, a small town in the southern part of the Brescia province, a factory is still active since the 1970s. In these areas recent research showed that pre-adolescent subjects exhibited impaired motor coordination and odor identification associated with elevated Mn exposure, and increased tremor intensity [9, 10]. In these circumstances we aimed to run a pilot brain imaging study to investigate effects of chemical toxicants as Mn on the central nervous system (CNS) of adolescents who spent their entire life in these geographical regions.

## Materials and Methods

### Participants

The pilot fMRI session included 14 pre-adolescents recruited in one of the three sites in the Province of Brescia. Ten of them (Mn-group) were from Bagnolo Mella (BM) and Valcamonica (VC), locations with history of manganese contamination and 4 from the Garda Lake (GL) which is an area free of Mn-contamination (controls). The mean age of the two groups was respectively 14.7 years (s.d. = 2.4) and 14.6 years (s.d. = 0.5) with 4 girls in the first group and 2 in the second. The 14 subjects enrolled were randomly selected from previous larger cohort created for a Phime/nih project headed by Roberto Lucchini. To select the 14 subjects we re-administered inclusion criteria questionnaire. Participants were at first identified through the local school district and recruited through school presentations. Participants were enrolled if they met the following inclusion criteria: were born in the respective area to a family who resided in the area for at least a generation, had lived in the study area since birth. Exclusion criteria included: known hand or finger motor deficits, visual deficits not adequately corrected and any history of neurological, metabolic, hepatic or endocrine diseases. Children were also excluded if they had a history of receiving parenteral nutrition that may cause Mn overload, were currently taking prescription psychoactive drugs or had known psychiatric disturbances. Written informed consent was obtained from parents and children. Study protocols were approved by institutional review board at the Ethical Committee of the Public Health Agency of Brescia. Extensive data on exposure assessment in these areas were published previously (Lucchini et al., 2012).

The olfactory function of each participant was assessed using the 12 items odor identification (Id score) Sniffin’ Sticks test [11] that has been demonstrated being the most sensitive olfactory test for olfactory impairments associated to Mn exposure, among the whole battery that includes threshold, discrimination and identification of odor sources [12]. Further, participants were trained to use the velopharyngeal closure technique, to avoid respiratory nasal air-flow, and breathe only with their mouth. This passive breathing technique prevents sniffing in response to odorous stimulation during the olfactory task.

### MRI acquisition

Before entering the scanner subjects completed an MRI exclusion criteria inventory. MRI data were collected at the Civil Hospital, University of Brescia using a 1.5T MRI unit [Aera, Siemens, Erlangen, Germany] equipped with a 12-channel phased array head coil. The MRI acquisition protocol included a Coronal T2 FSE (TR/TE 5000/102 slice thickness 2 mm, no gap) use for the volumetric measurement of the olfactory bulb; a time series of 2D echo planar imaging (EPI) (TR/TE 2500/50 ms, 28 axial slices, 3.3 mm thickness, 1.1 mm inter-slice gap, matrix size 64x64) sensitive to blood-oxygen-level dependent (BOLD) contrast to register the functional magnetic resonance imaging (fMRI) volumes; and a high resolution 3D magnetization-prepared rapid gradient echo (MPRAGE) T1-weighted scan (TR/TE 2040/3.08 ms, 256 mm FOV, 320*320 matrix, 144 sagittal slices, 1.0 mm3 voxel sixe) important for segmentation and normalization of the young brains with the standard MNI (Montreal Neurological Institute)-space.

### fMRI paradigm

The fMRI, olfactory stimulation consisted of 12 alternating task-rest blocks of 25 seconds, 10 volumes/block (120 volumes) for a total acquisition time of 5 minutes. Before and after the fMRI session, the subjects were asked to rate the intensity of the stimuli on a scale ranging from 0 (extremely weak) to 10 (extremely strong).

### Stimulus conditions and Odorant delivery system

The odors were delivered by means of an MRI compatible computer controlled olfactometer [13], where we could define parameters such as the pulse duration (1s) and the inter-stimulus interval within the block (2s) for the entire sequence. The paradigm was built up by block of pulsed odor with the above mentioned conditions (ON-block) interleaved by block of odorless air flow (control condition also called OFF-block). The output flow was set at 2 L/min. As odor stimulus we use pure phenyl ethyl alcohol (PEA–Sigma Aldrich Chem. Steinheim, Germany), a rose like smell. The subjects were instructed to breath only through the mouth in order to prevent undesired effects of sniffing (see above). To further control the sniffing rate we used a respiratory sensor in front of the nose of the subject and compatible with the scanner environment.

### fMRI and psychophysics data analysis

The fMRI data were analyzed with SPM8-software (Statistical Parametric Mapping; Welcome Department of Cognitive Neurology, London, UK) implemented in Matlab R2013a (Math Works Inc., MA, USA). Spatial pre-processing involved realignment to minimize movement effects, co-registering of functional- and structural images and image normalization in a stereo-tactic space. Finally all the functional data were smoothed by means of a 7*7*7 mm^3^ “full width at half maximum” Gaussian kernel in order to improve the signal-to-noise-ratio and suppress residual differences between subjects. Subsequently, preprocessed functional data were modeled in a GLM first level analyses for each single-subject with canonical hemodynamic and its derivative set functions available in SPM. To correct for residual movement artefacts, movement parameters were included in the matrix design as regressors. Two out of the 14 volunteers were excluded from the analysis; one for movement artefacts and the second for acute sinusitis at the time of the experiment that obstructed the odorant to reach the olfactory cleft and so to perceive the stimulus. The group analysis was carried on by a random-effect analysis (RFX) based on the ON minus OFF contrast assessed at the first level and modeled in an ANOVA one way (one stimulus condition, two groups).

Due to the small size of the sample and to the general interest of the pilot study the results were first assessed at whole group level (exposed+control). Secondly to have a more general overview of the differences between the two groups we evaluated also the comparison (control-exposed). This was done in awareness that the matrix design is strongly unbalanced. However, the parametric statistics was thought to be robust enough to generally indicate a trend of a more appropriate sample.

Finally the change of %BOLD was evaluated at the single subject level in a volume of interest (VOI) located in the left and right olfactory tract and primary olfactory cortex.

### Bulb volumetry analysis

The volumetric measurements of the right and left OB were performed by manual segmentation of the coronal slices through the OBs using the AMIRA 3D visualization and modeling system (Visage Imaging, Carlsbad, USA). As first shown by Yousem and colleagues [14] this procedure is highly reliable being able to obtain coefficients of correlation for repeated measurements by a single observer greater than .92 while for measurements across observers the coefficients were event greater (.96). The segmentation on the T2-weighted MRI of the 14 subjects acquired in Brescia was done by one observer twice and in a blind way in respect to the set of the geographical location of origin, and therefore to the exposure classification of each subject. The measure of the OB for each subject was then evaluated by the average of the two.

Further statistical analyses were carried out by means of SPSS 19.0 (SPSS Inc., Chicago, IL, USA). To test the hypothesis we used the Mann-Whitney U test with the level of significance set at 0.05.

## Results

### Odor identification (Id score)

The VC/BM group showed a Id score average of 10 (s.d. = 1) on a maximum of 12points; the control (GL-group) showed an Id score average of 9 (s.d. = 2). The M-W U test revealed no significant differences between the two groups (M-W Utest = 9.00; Z = -0.864; p = .387).

### Ratings

**Fig 1:**
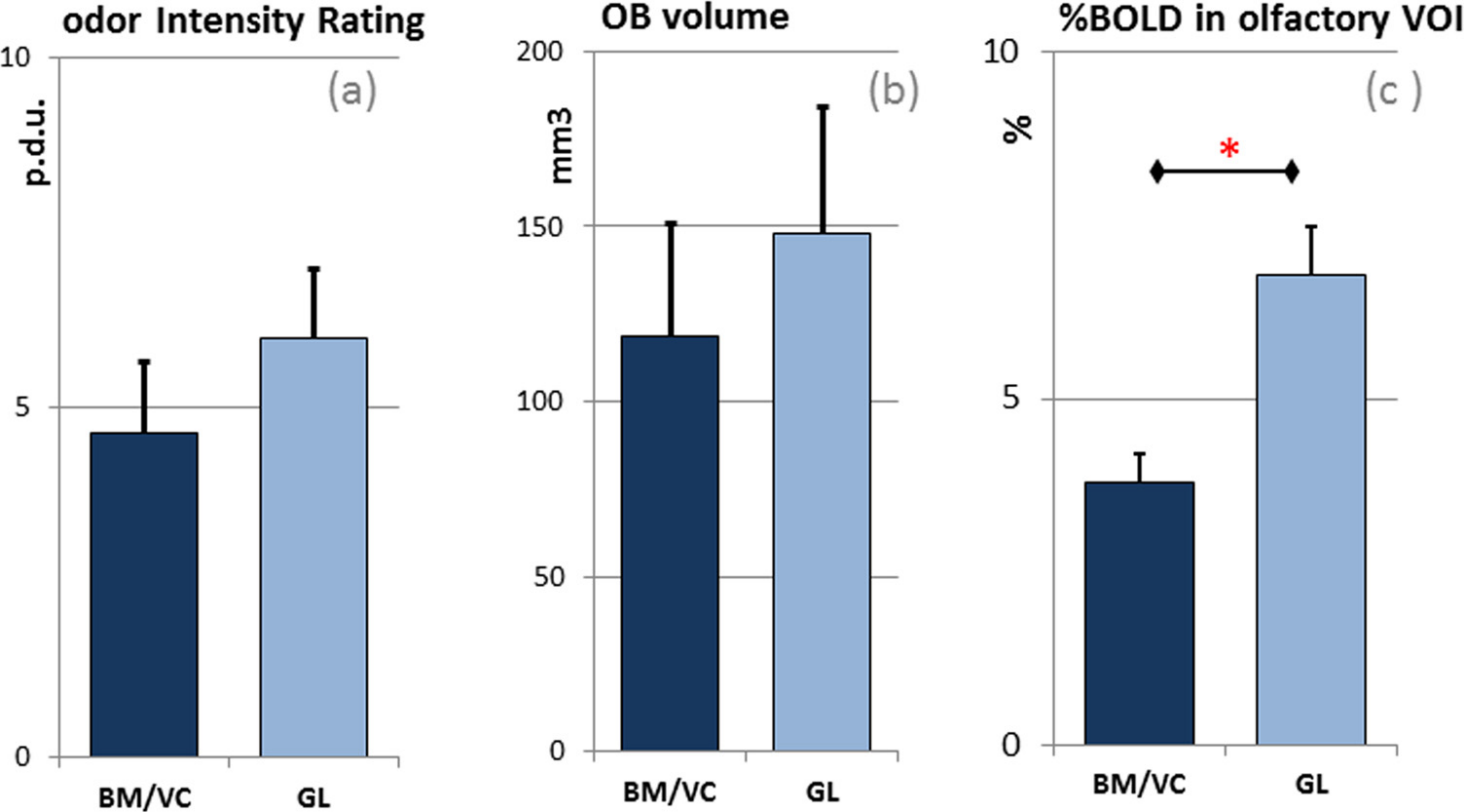
Specific response of the two groups BM/VC (manganese exposed) and GL (control) for (a) odor intensity rating evaluated during the fMRI session in procedure definition units (p.d.u.); (b) volume of the olfactory bulb in mm^3^; (c) Estimated sustained response to olfactory stimulation in the specific volume of interest (VOI) including olfactory tract and primary olfactory cortex in perceptual variation of the BOLD signal. The * in the figure corresponds to significant level p < .05.

The VC/BM group rated the intensity of the stimulus an average with 4.7 (s.d. = 2.1) and the GL group with an average value of 6.0 (s.d. = 1.7), which were not statistically different (M-W Utest = 9.5; Z = -0.937; p = .349) (Fig 1A).

### fMRI with olfactory task

**Fig 2:**
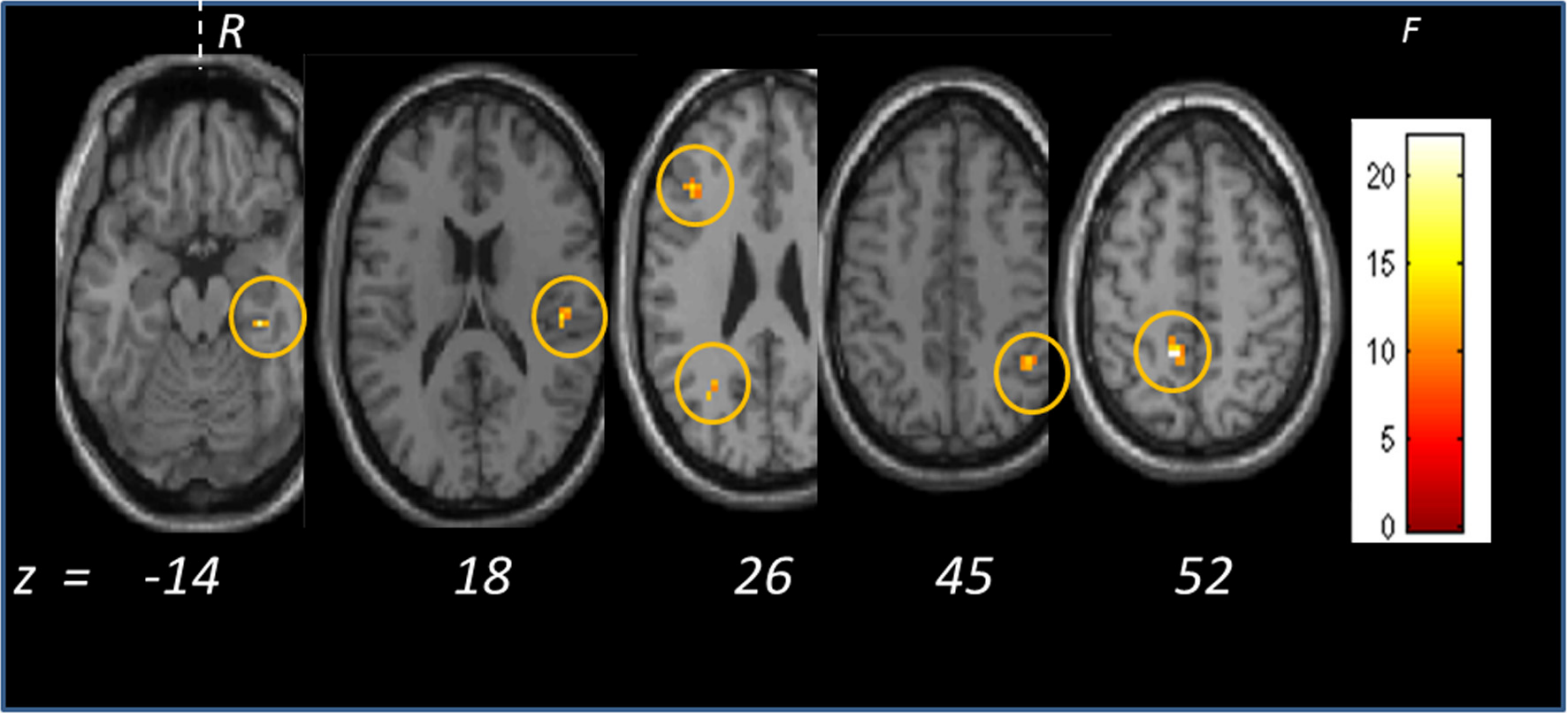
General effect of the odor stimulation on the group of teenagers from the three sites in Italy. The olfactory stimulation induced activation in scattered areas including precuneus, parahippocampal gyrus, insula, middle frontal and temporal gyrus and inferior parietal lobule. The statistical F-value is color coded (F[2,10], p < .005).

As **general effect of interest** (F[2,10], p < .005) independent on the geographical location of origin, the olfactory stimulation induced activation in scattered areas including precuneus, parahippocampal gyrus, insula, middle frontal and temporal gyrus and inferior parietal lobule (Table 1, Fig 2).

**Table 1:** General effect of the odor stimulation on the group of 12 teenagers.

*# of voxels*	*F*	*Z*	*p(unc)*	*Talairach (mm)*			*Brain areas*		
				*X*	*Y*	*Z*	*lobes*	*lables*	
**11**	22.30	3.53	0.000	12	-42	52	parietal	**precuneus**	
**6**	18.60	3.33	0.000	33	-28	-14	limbic	**parahippocampal gyrus**	
**6**	13.87	3.01	0.001	48	-22	18	limbic	**insula**	
**8**	13.66	2.99	0.001	-40	29	21	frontal	**middle frontal gyrus**	**DL- PFC**
**6**	12.61	2.90	0.002	-34	-62	28	temporal	**middle temporal gyrus**	
**8**	12.60	2.90	0.002	42	-45	45	temporal	**inferior parietal lobule**	

The table shows for each maximum activation voxels in Talairach space the relative F-value, Z-value, the p-value, and the number of voxel in the cluster.

The **differences** between the Mn-exposed subjects (9 subjects) and the controls (3 subjects) in both direction was assessed by means of a 2-sample t-test (T[10], p < .01) on the whole brain. We found that the response of the control was stronger in areas typically associated with the secondary olfactory cortex, like middle frontal gyrus left and right, cerebellum and supplementary motor areas (SMA) (Table 2). The last two areas are eventually related to the intention to sniff.

**Table 2:** Differences between the control versus the exposed subjects.

# of voxel	T	Z	p(unc)	Talairach (mm)			*Brain areas*	
				x	y	z	lobes	lables
**10**	5.44	3.63	0.000	-30	-64	57	parietal	**superior parietal lobule**
**35**	5.13	3.51	0.000	33	10	46	frontal	**middle frontal gyrus**
**11**	4.28	3.15	0.001	-21	-52	-23	cerebellum	**anterior lobe**
**13**	4.16	3.10	0.001	-46	22	19	frontal	**middle frontal gyrus**
**10**	3.94	2.99	0.001	9	-48	45	parietal	**precuneus**
**12**	3.91	2.97	0.001	36	51	7	frontal	**middle frontal gyrus**

The table shows for each maximum activation voxels in Talairach space the relative T-value, Z-value, the p-value, and the number of voxel in the cluster.

The reverse contrast (Mn-exposed>control) revealed no supra-threshold cluster, indicating a generally lower response of the exposed Mn group.

**Fig 3:**
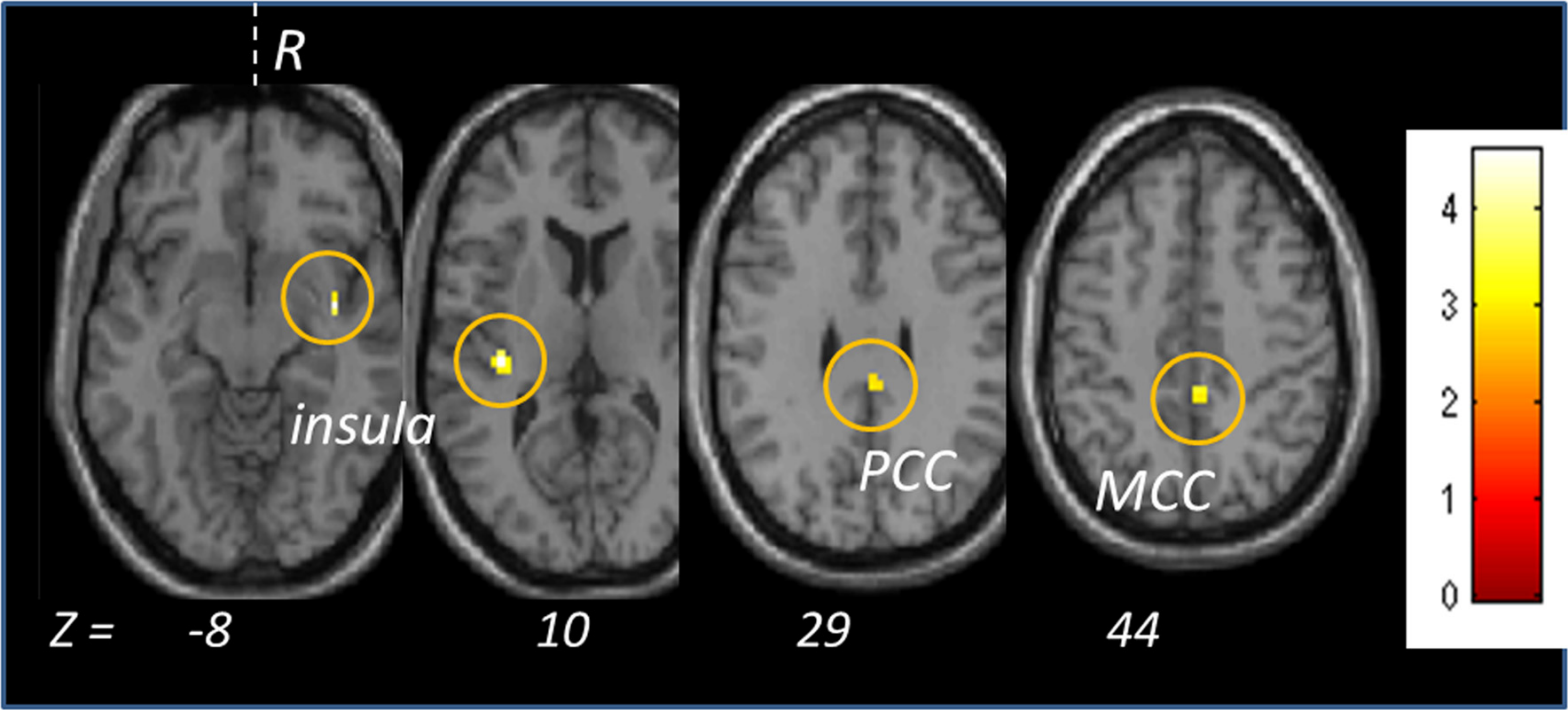
Differences between the control versus the exposed subjects in the limbic system. Circles are regions of activity differences following olfactory stimulation comparing the GL (referents) versus the Mn-exposed BM+VC subjects in limbic areas. The statistical T-value is color coded. R: right, PCC: posterior cingulate cortex, MCC: middle cingulate cortex.

In order to explore whether Mn exposure modifies the olfactory-limbic system network, we performed a SPM analysis on a region of interest (ROI) including limbic areas (olfactory tract, piriform, entorhinal, amygdala, insula, cingulate gyrus, hippocampus, parahippocampal gyrus). The results showed that the control subjects responded to the odors more strongly than the Mn-exposed subjects respectively in the left and right insula and middle and posterior cingulate (Table 3, Fig 3). The reversed contrast (Mn-exposed>control) didn’t show any activity in this specific brain ROI suggesting a specifically reduced response in the limbic system associated with residence in BM/VC.

**Table 3:** Differences between the control versus the exposed subjects in the limbic system.

*# of voxels*	*T*	*Z*	*p(unc)*	*Thalairach (mm)*			*limbic system*
				*x*	*y*	*z*	
**3**	4.61	3.30	0.000	40	-4	-8	**insula**
**8**	4.54	3.27	0.001	-36	-24	10	**insula**
**8**	3.29	2.64	0.004	3	-34	44	**precuneus/mid. cingulate c.**
**4**	2.86	2.39	0.008	3	-32	29	**posterior cingulate cortex**

The table shows for each maximum activation voxels in Talairach space the relative T-value, Z-value, the p-value, and the number of voxel in the cluster.

To directly compare the hemodynamic response in olfactory relevant areas between the Mn-exposed and non-exposed subjects we extracted the percent variation of the BOLD (%BOLD) signal in two symmetric volumes of interest (VOI) centered respectively in the left and right olfactory tract [(±5, 20,-11) MNI stereotactic space; volume box of 4x16x20 mm^3^] and including the anterior and the posterior piriform cortex, the putative primary olfactory cortex. From that the estimation of the percent variation of the BOLD signal was evaluated in comparison to the baseline derived from the off-block. The mean % BOLD in the BM/VC group was .038 (s.d. = ±.005), while it was .068 (s.d. = ±.039) in the control group (Fig 1C) (Mann-Whitney test: U = 1.500; Z = -2.231; p = .026).

### Olfactory bulb volumetry

Average olfactory bulb volume, left and right, was for VC/BM averaged out at 118 (s.d. = ± 34) mm^3^ and for GL 148 (s.d. = ± 27) mm^3^ (Fig 1B). A Mann-Whitney test did not show any significant difference between the two group (M-W U test = 9; Z = -1.189; p = 0.234).

## Discussion

As already demonstrated in preview works olfactory function can be altered if humans are exposed for a relatively long time to elevated levels of Mn. In the present study, changes in olfactory functions were largely assessed with psychophysical tests like the response to standardized odor sources (e.g. Sniffin’ Sticks test) [9, 10]. Moreover using specific features like discrimination, identification and threshold of odors test sniffin’ sticks, Guarneros and colleagues in a recent study [12] indicate that olfactory function relying to a greater extent on memory and cognition could be also affected since manganese may reach deep brain structures through trans-synaptic transport from the intranasal olfactory epithelium. To explore directly these directions at the CNS level we implemented a pilot brain imaging experiment where, together with the olfactory psychophysical response, we could investigate olfactory brain related areas on both functional and structural levels.

We found a reduced hemodynamic function in the relevant olfactory areas, namely orbitofrontal cortex and piriform cortex, in the exposed group (VC/BM group) compared with the sample population in a non-exposed area (GL controls). Moreover the statistical parametrical mapping of the reference group compared to the VC/BM subjects was significantly more activate in brain areas typically associated with olfactory function, like the middle frontal gyrus, left and right, and the cerebellum. The reverse contrast (i.e., VC/BM vs. GL) revealed no supra-threshold cluster, indicating a generally lower sensory-odor response in the exposed -group. This is also in agreement with [12] where the authors deduced a more pronounced effect on the central nervous system than just peripheral.

The same occurred when we restricted the observation in ROI limbic areas, a complex of brain regions commonly associated with regulation of emotion triggered form environmental stimuli and–to some degree—also linked to odor perception [15, 16], where the results showed a reduction of activity for the BM/VC group. Now it is well known that adolescence is a period of immense developmental changes and brain re-organization associated with cognitive and emotional functions [17, 18]. This, considered together with our fMRI preliminary results, suggests that the effect of Mn exposure can have major consequences also on the human behavioral later in life. Important here is to notice that the data size of our sample reduce the possibility to infer directly on them, moreover statistical parametrical mapping of fMRI data are prone to false discovery rate due to large numbers of voxels involved in the statistics.

The psychophysical assessment of the Id scores as well as the OB volume was not significantly different among the two groups. However we observed a tendency for a decreased response in the exposed group (Fig 1A and 1B). We strongly believe that this trend can be significant with a larger sample size. To verify this hypothesis we added to the control group a dataset with identical data characteristics and population matched for age (t[30] = -0.272, p = 0.7988) and gender (χ^2^(1, n = 32).002, p = .961). The sample included 18 pre-adolescent subjects (mean age 14.4, s.d. = 2.8, 7 woman) with no history of manganese exposure and normal olfactory functions (Id average score/max score = 11.1/12; s.d. = 0.9). We measured the olfactory bulb volume (using the identical procedure as for the other participants) with a mean of 161 mm^3^ (s.d. = 17 mm^3^). Then we merged the 18 non-exposed subjects with the control group and reapplied the Mann-Whitney U test to reinvestigate the hypothesis above mentioned. The results indicated a significantly reduced OB volume and decreased Id scores in the VC/BM group in comparison to the non-exposed group, with the following results respectively: M-W U test = 33.0; Z = -2.58; p = 0.01; and M-W U test = 51.5; Z = -2.01; p = 0.044.

## Conclusions

In conclusion our results suggest that environmental exposure to Mn for long time span in the first stage of life, from childhood to pre-adolescence, can jeopardize olfactory functions not only in terms of a reduced subjective odor sensitivity, as it has been shown in psychophysical studies [9, 10] and confirmed here, but also can reduce the volume of olfactory eloquent brain structures like the OB with consequently disturbed olfactory functions (decreased BOLD signal).

As a second stage, Mn exposure, can affect also the functionality of the limbic system that in the adolescence undergoes to massive pruning of synapses to form the mature system, essential to control emotions. Therefore, these preliminary data strongly support the original idea of environmental Mn exposure toxicity in childhood and pre-adolescence and indicate valid methods to further investigate this important issue.
